# Management of Ruptured Subcapsular Liver Hematoma as a Result of Hemolysis, Elevated Liver Enzyme, and Low Platelet Syndrome in a Rural Facility

**DOI:** 10.7759/cureus.33852

**Published:** 2023-01-16

**Authors:** Audrey Marinelli, Jennifer Hill

**Affiliations:** 1 Department of Obstetrics and Gynecology, University of Connecticut Health, Farmington, USA; 2 Prenatal Testing Center, Division of Maternal-Fetal Medicine, Department of Obstetrics and Gynecology, Hartford Hospital, Hartford, USA

**Keywords:** hemolysis elevated liver enzymes and low platelets, emergency obstetric care, subcapsular hepatic hematoma, argon beam, subcapsular hepatic rupture, hellp, pre-eclampsia

## Abstract

Subcapsular liver hematoma is a rare complication of hemolysis, elevated liver enzyme, and low platelet (HELLP) syndrome. This case depicts a previously healthy 27-year-old primigravida at 39 weeks' gestation who presented with severe abdominal pain and was diagnosed with HELLP syndrome based on vital signs and laboratory values. While arranging transport to a regional perinatal care facility she became acutely unstable with maternal hypotension and resultant fetal bradycardia. An emergent cesarean section was performed and a ruptured subcapsular liver hematoma was diagnosed intraoperatively. She was successfully managed with supportive care and surgical intervention and was discharged from the hospital on postoperative day 6. Here we review the indications and methods of conservative and surgical management of subcapsular liver hematoma as a result of HELLP syndrome.

## Introduction

Hemolysis, elevated liver enzyme, and low platelet (HELLP) syndrome are relatively common, occurring in 0.5%-0.9% of pregnancies, and up to 20% of cases of pre-eclampsia with severe features [1]. The criteria most frequently used to diagnose HELLP syndrome include aspartate aminotransferase (AST) and alanine aminotransferase (ALT) ≥ 100 IU/L or twice the upper limit of normal; platelet count < 100 × 10^9^/L; and lactate dehydrogenase (LDH) > 600 units/L [2].

Although rare, one of the most severe manifestations of HELLP syndrome is the formation of a subcapsular liver hematoma, occurring between 1:45,000 and 1:225,000 pregnancies [3]. The most catastrophic sequelae of HELLP syndrome are the rupture of the subcapsular hepatic hematoma, and maternal mortality has been reported to be as high as 17%-59% [4]. This may present as severe epigastric or right upper quadrant pain, nausea, vomiting, hypotension, or hemorrhagic shock [3]. We present a case of ruptured subcapsular hematoma as a result of HELLP syndrome that required surgical intervention.

This article was previously presented as a meeting poster at the 2022 ACOG District 1 Annual Meeting on October 28, 2022.

## Case presentation

This is a 27-year-old primigravida at 39 weeks' gestation with a history of gastroesophageal reflux disease (GERD) who presented to labor and delivery with epigastric and right upper quadrant abdominal discomfort. She had worsening nausea and vomiting and was also found to have new onset hypertension. She had been seen earlier that day and at 38 weeks' gestation with vague epigastric pain attributed to GERD. During both of these encounters she was normotensive, had a normal abdominal exam, and felt improved with antacids. No labs were obtained during these two previous encounters. She had no other past medical or surgical history. A pre-eclampsia panel including complete blood count (CBC), comprehensive metabolic panel (CMP), LDH, and uric acid laboratory values was obtained due to her hypertension and are displayed in Table 1.

**Table 1:** Admission vital signs and laboratory values

Measurement	Value(s)	Reference Ranges in Pregnancy
Blood Pressure	121-151/82-98 mmHg	<140/90 mmHg
Pulse	88-97 bpm	60-100 bpm
Hemoglobin	11.6 g/dl	11.0-13.5 g/dl
Platelets	70 × 10^9^/L	150-400 × 10^9^/L
Aspartate Aminotransferase	550 IU/L	7-55 IU/L
Alanine Aminotransferase	502 IU/L	8-48 IU/L
Creatinine	0.8 mg/dL	0.4-0.8 mg/dL
Lactate Dehydrogenase	619 units/L	105-333 units/L
Uric Acid	6.4 mg/dL	3.5-7.2 mg/dL
Protein:Creatinine Ratio (Urine)	0.325	<0.300

All other lab values were within normal limits. Her fetal heart rate tracing was reassuring. The patient was diagnosed with HELLP syndrome, started on magnesium sulfate for seizure prophylaxis, and transport was arranged to a regional perinatal center given her thrombocytopenia and lack of adequate blood products at the initial facility. Shortly after admission, and before she was transported, she became acutely unstable with excruciating pain and significant hypotension (blood pressures of 60/30s mmHg) and fetal bradycardia requiring emergent delivery via primary cesarean section. 

Upon entering the abdomen, massive hemoperitoneum was noted with active bleeding from the upper abdomen. After delivery of the neonate and closure of the hysterotomy, the skin incision was extended into a midline vertical T-shaped incision. The bleeding could not be adequately visualized and due to the patient’s instability, the abdomen was packed with moist laparotomy pads by the obstetrics provider and an intraoperative consult from general surgery was requested. The massive transfusion protocol was initiated with packed red cells and fresh frozen plasma (FFP). The magnesium sulfate infusion was paused due to hypotension. When the general surgery team arrived, an upper abdominal survey noted a normal appearing spleen with active bleeding noted from the liver capsule. The decision was made to re-pack the abdomen with laparotomy pads, close with an AbThera (3M, Saint Paul, MN, USA) device, and transfer to the ICU while arranging transport to the regional perinatal center via helicopter. The total blood loss at this point was approximately 2.5L and prior to transfer the patient had received two units of packed red blood cells, three units of FFP, and two units of platelets.

When she arrived at the regional perinatal center, she was coagulopathic with a fibrinogen of 215 (normal third trimester value is over 300), partial thromboplastin time (PTT) of 20, and vital signs consistent with stage three hemorrhagic shock. She was aggressively supplemented with intravenous fluids and a total of four units of packed red blood cells, five units of FFP, and four units of platelets were transfused with resolution of her hypotension and coagulopathy. Magnesium therapy was restarted at this time and continued through 24 hours postpartum. On postpartum day 1, despite massive transfusion protocol and resolution of her coagulopathy, she had severe anemia with her hemoglobin down-trending from 12.5 g/dL > 6.5 g/dL. Due to the suspicion of ongoing bleeding from the liver capsule, she underwent a re-exploration through the midline vertical incision with trauma surgery and the obstetrics team.

**Figure 1:**
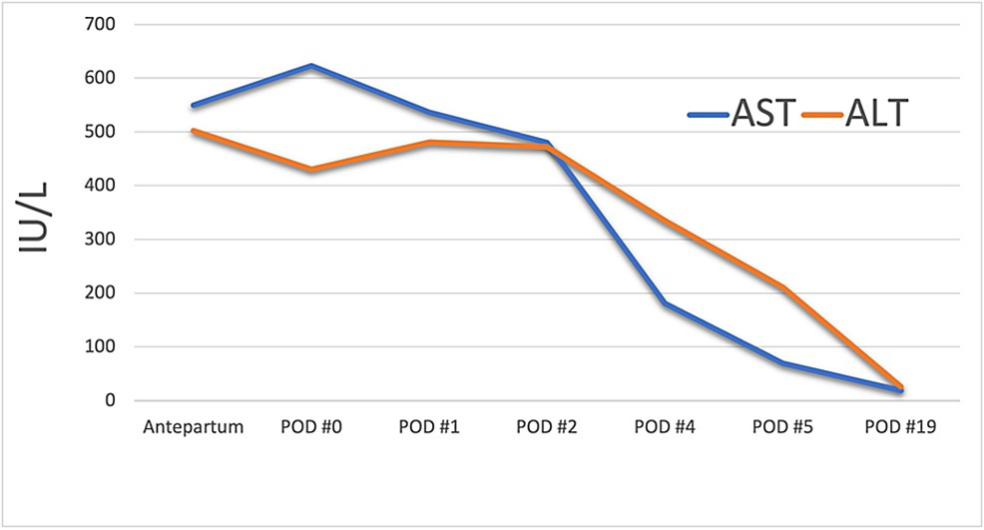
Resolution of transaminitis AST - aspartate aminotransferase, ALT - alanine aminotransferase, POD - post operative day

**Figure 2:**
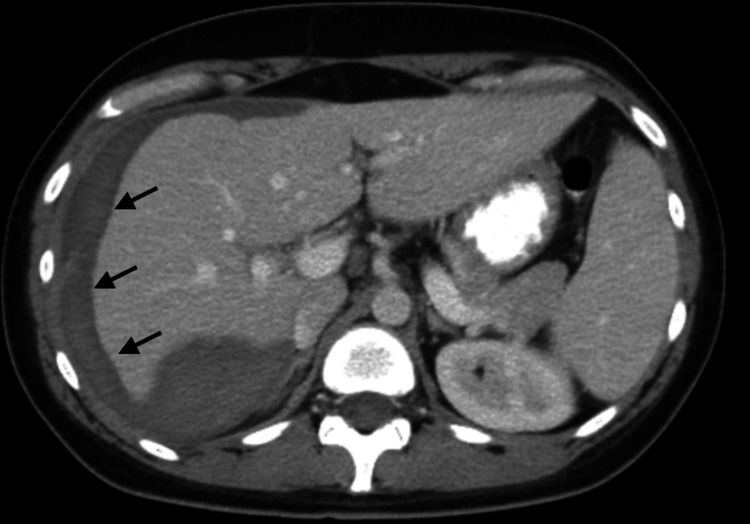
Computerized tomography scan on postpartum day 14 showing the persistence of hematoma Arrows showing location of subcapsular liver hematoma

A large, denuded area was noted on the surface of liver segments six, seven, and eight with general oozing. The margins of the capsule were not affixed to the liver, were elevated, and contained intact hematoma. Argon beam was used to coagulate the denuded, oozing surface. Evarrest (Ethicon, Cincinnati, OH, USA) fibrin sealant patches were placed over the liver capsule and pressure applied with multiple laparotomy pads. Post-operatively, her labs remained stable and on postpartum day three she underwent the final re-exploration where the liver edge was noted to be hemostatic without applying pressure and her abdomen was closed. The remainder of her recovery was uneventful. Her transaminitis and thrombocytopenia continued to improve (Figure 1) and she was discharged on postpartum day six in stable condition with nifedipine 30mg daily. She was seen on postpartum day 14 with resolution of her transaminitis but worsening right upper quadrant pain. There was a concern for hepatic abscess given her worsening pain, however computerized tomography (CT) scan showed the remaining subcapsular liver hematoma with no evidence of abscess (Figure 2).

 

## Discussion

The pathogenesis of subcapsular liver hematoma in HELLP syndrome is not completely understood [5]. It is theorized that pre-eclampsia can predispose to hepatic infarction or vasospasm. When neovascularization occurs in the hepatocytes, these new fragile vessels are prone to rupture during hypertensive events, leading to a hematoma. As the liver capsule is thin and friable, relatively small traumas or movements can lead to hematoma rupture [6,7].

Prompt diagnosis and management of HELLP syndrome are imperative, as patients with HELLP syndrome have been shown to be at increased risk of maternal morbidity compared to pre-eclampsia with severe features, including the need for blood transfusions, disseminated intravascular coagulation (DIC), wound hematoma or infection, and acute renal failure [8].

As in this case, the diagnosis of HELLP syndrome is often delayed. Up to 15% of cases of HELLP syndrome lack hypertension and proteinuria, obscuring and delaying diagnosis [9]. In addition, it is difficult to predict which patients with HELLP syndrome may develop a hepatic hematoma, as the degree of transaminitis does not correlate with evidence of hematoma on imaging [10]. However, most patients with a subcapsular hematoma will be symptomatic, with 90% of patients experiencing right upper quadrant or epigastric pain and 50% complaining of nausea and vomiting [11]. For this reason, all gravid patients complaining of right upper quadrant or epigastric pain should receive imaging to rule out liver hematoma, and providers should not be reassured by normal laboratory values or lack of hypertension.

Management

The management of liver hematoma secondary to HELLP syndrome depends on if the hematoma has ruptured as well as the hemodynamic stability of the patient [5]. For patients with an unruptured hematoma who are hemodynamically stable, conservative therapy has been advocated to avoid iatrogenic liver injury with more invasive management options [12]. Supportive therapy including fluid resuscitation and transfusions of blood products to correct anemia and coagulopathy is often necessary. Patients should be re-imaged periodically to assess for expansion of the hematoma. Bedside ultrasound and computerized tomography (CT) scans are preferred due to their ease of use and quick diagnosis.

Hemodynamic instability from a ruptured liver hematoma requires emergent surgical intervention with a multi-disciplinary team including surgeons familiar with liver trauma [13]. In addition, patients should receive aggressive resuscitation with a massive transfusion protocol. Rarely, patients with ruptured subcapsular hematomas are hemodynamically stable, and case reports have described conservative management in these patients [14]. Several surgical methods to control hemorrhage have been described, and often successful management requires multiple methods. As seen in this case, hematoma formation is most likely to affect the right lobe of the liver [15]. In a recent literature review, packing the abdomen to apply pressure to the liver capsule was the most frequent intervention with an 82% survival in a small case series [16,17]. In rural facilities without access to trauma surgeons, obstetricians may need to provide the initial surgical management of ruptured subcapsular hematomas as a result of HELLP syndrome, as seen in this case. In addition to the transfusion of blood products, packing the abdomen with laparotomy sponges is a simple yet effective way to control bleeding and provide the hemodynamic stability required to transport these patients to regional perinatal care centers where more definitive management can be pursued. Other interventions include argon beam coagulation, drainage of the hematoma, and suture of omentum or mesh to reinforce the disrupted capsule [12]. Management with hepatic lobectomy is associated with high mortality and should therefore be avoided [16]. Interventional radiology-guided hepatic artery embolization has been used alone or in combination with surgery with good outcomes [5,18]. In dire cases where hemorrhage is not controlled by the above measures or there is significant liver necrosis or liver failure, a liver transplant may be necessary [19].

## Conclusions

Although rare, clinicians must have a high suspicion for subcapsular liver hematoma related to HELLP syndrome in gravid patients complaining of right upper quadrant or epigastric abdominal pain. Lack of transaminitis, proteinuria, or hypertension does not rule out the possibility of subcapsular liver hematoma. Imaging is recommended to rule out hematoma formation in patients with ongoing pain or discomfort, especially if accompanied by nausea and vomiting. Management of subcapsular liver hematoma in HELLP syndrome depends on the hemodynamic stability of the patient and presence of rupture. In patients who are unstable, emergent surgical management is most often necessary, while patients who are hemodynamically stable may be managed conservatively. All patients benefit from increased surveillance and transfusions of blood products to correct resultant anemia and coagulopathy.
